# First case of hemorrhoidal carcinoma in a fistulotomy scar: successful per-anal endoscopic myectomy despite severe fibrosis

**DOI:** 10.1055/a-2721-9065

**Published:** 2025-11-14

**Authors:** Xiaona Shao, Naoya Toyoshima, Toshihiro Haga, Mitsunori Kusuhara, Yukihide Kanemitsu, Jianwei Shen, Yutaka Saito

**Affiliations:** 1 74634 Department of Gastroenterology, Ningbo Medical Center Lihuili Hospital, Ningbo, China; 2 68380 Department of Endoscopy, National Cancer Center Hospital, Tokyo, Japan; 3 13874 Department of Pathology, National Cancer Center Hospital, Tokyo, Japan; 4 12912 Department of Gastroenterology and Hepatology, Kyorin University, Tokyo, Japan; 5 Department of Colorectal Surgery, National Cancer Center Hospital, Tokyo, Japan

Rectal endoscopic submucosal dissection (ESD) offers a minimally invasive, function-preserving alternative to radical surgery, allowing en bloc resection and precise histopathological assessment. However, severe submucosal fibrosis with muscle retraction can significantly hinder complete resection. Following Rahni et al.’s pioneering description of per-anal endoscopic myectomy (PAEM) for R0 resection of such lesions 1 , we report the first case of hemorrhoidal carcinoma arising in a post-fistulotomy scar successfully treated using this technique.

A 70-year-old man with a history of anal fistula surgery presented with occult blood in stool. Colonoscopy revealed a 10-mm type 0-Is lesion (1 cm from the dentate line) with the JNET 2B/type IV pit pattern ( Fig. 1 ). Initial ESD at the referring hospital was aborted due to suspected muscular invasion (biopsy: well-differentiated adenocarcinoma). At our center, image-enhanced endoscopy confirmed Tis-T1a carcinoma (JNET 2B/type IV) despite marked fibrosis ( Fig. 2 ). PAEM achieved en bloc R0 resection ( Fig. 3 ); histopathology confirmed hemorrhoidal adenocarcinoma, an exceedingly rare malignancy ( Fig. 4 ). No recurrence was noted at 6-month follow-up ( Fig. 5 ).

**Fig. 1:**
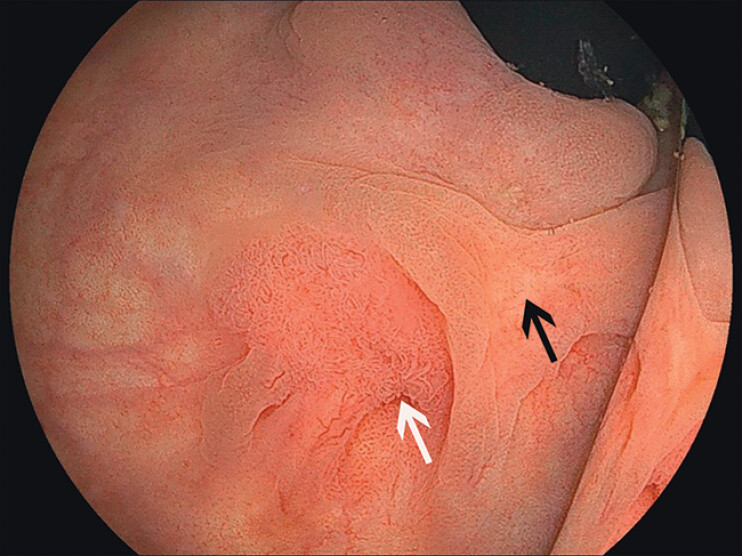
Endoscopic image (Rb): the 10-mm 0-Is lesion near anal verge (white arrow: lesion; black arrow: scar).

**Fig. 2:**
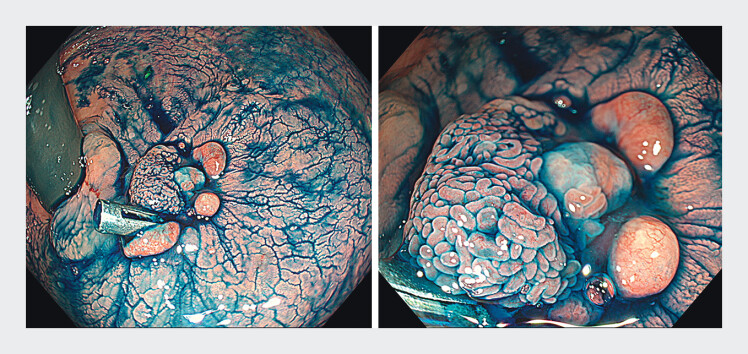
Indigo carmine dyeing shows the flat elevated lesion in the post-fistulotomy scar and assessed as Tis-T1a.

**Fig. 3:**
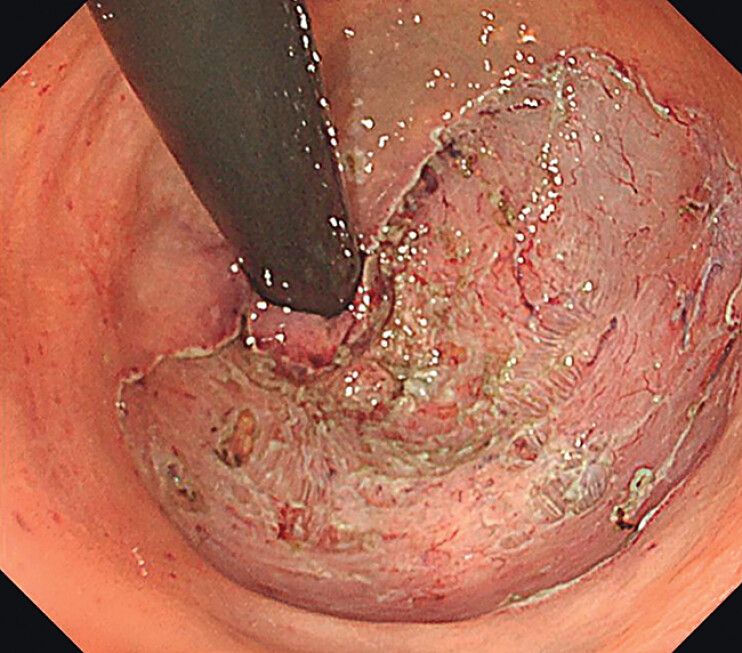
En bloc resection of the fibrotic lesion via PAEM. PAEM, per-anal endoscopic myectomy.

**Fig. 4:**
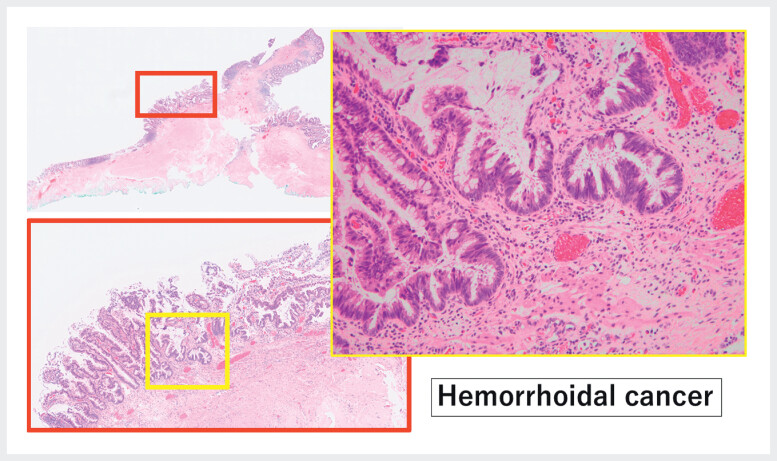
11 × 10 mm specimen (pTis, Vienna 4.2) showing mucosal adenocarcinoma involving fistula orifice.

**Fig. 5:**
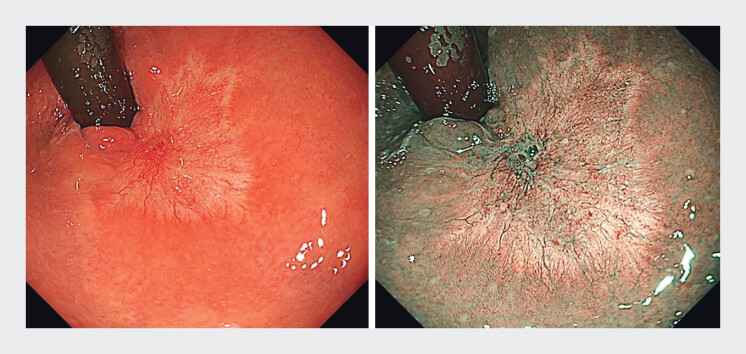
Mucosal healing at 6-month follow-up; no recurrence.

This case highlights PAEM’s safety and efficacy for severely fibrotic lesions ( Video 1 ). Fistula-associated carcinomas occur in ~1% of chronic anal fistulas 2 , often with delayed diagnosis. To our knowledge, this is the first reported case of endoscopic resection of hemorrhoidal carcinoma arising from a fistulous tract.

PAEM employs dual-tunnel circumferential dissection to isolate fibrotic lesions, transecting the inner circular muscle while preserving the outer longitudinal muscle for R0 resection. PAEM, per-anal endoscopic myectomy.Video 1

PAEM enabled (1) complete resection, including fistula-involved tissue and (2) dissection while avoiding submucosal fibrosis. Key points: (1) magnified endoscopy with pit pattern analysis is essential for accurate staging in distorted anatomy; (2) multidisciplinary management is crucial; (3) chronic anorectal wounds warrant surveillance due to malignant potential.

PAEM is an effective salvage strategy for ESD-ineligible fibrotic lesions 3 .

Endoscopy_UCTN_Code_CCL_1AD_2AB

## Notes

Endoscopy E-Videos https://eref.thieme.de/e-videos
*E-Videos* is an open access online section of the journal *Endoscopy* , reporting on interesting cases and new techniques in gastroenterological endoscopy. All papers include a high-quality video and are published with a Creative Commons CC-BY license. Endoscopy E-Videos qualify for HINARI discounts and waivers and eligibility is automatically checked during the submission process. We grant 100% waivers to articles whose corresponding authors are based in Group A countries and 50% waivers to those who are based in Group B countries as classified by Research4Life (see: https://www.research4life.org/access/eligibility/ ). This section has its own submission website at https://mc.manuscriptcentral.com/e-videos .
